# Therapeutic Targeting of STAT3 (Signal Transducers and Activators of Transcription 3) Pathway Inhibits Experimental Autoimmune Uveitis

**DOI:** 10.1371/journal.pone.0029742

**Published:** 2012-01-05

**Authors:** Cheng-Rong Yu, Yun Sang Lee, Rashid M. Mahdi, Narayanan Surendran, Charles E. Egwuagu

**Affiliations:** 1 Molecular Immunology Section, National Eye Institute, National Institutes of Health, Bethesda, Maryland, United States of America; 2 Orchid Research Laboratories Limited, Chennai, India; Emory University, United States of America

## Abstract

Mice with targeted deletion of STAT3 in CD4^+^ T-cells do not develop experimental autoimmune uveitis (EAU) or experimental autoimmune encephalomyelitis (EAE), in part, because they cannot generate pathogenic Th17 cells. In this study, we have used ORLL-NIH001, a small synthetic compound that inhibits transcriptional activity of STAT3, to ameliorate EAU, an animal model of human posterior uveitis. We show that by attenuating inflammatory properties of uveitogenic lymphocytes, ORLL-NIH001 inhibited the recruitment of inflammatory cells into the retina during EAU and prevented the massive destruction of the neuroretina caused by pro-inflammatory cytokines produced by the autoreactive lymphocytes. Decrease in disease severity observed in ORLL-NIH001-treated mice, correlated with the down-regulation of α4β1 and α4β7 integrin activation and marked reduction of CCR6 and CXCR3 expression, providing a mechanism by which ORLL-NIH001 mitigated EAU. Furthermore, we show that ORLL-NIH001 inhibited the expansion of human Th17 cells, underscoring its potential as a drug for the treatment of human uveitis. Two synthetic molecules that target the Th17 lineage transcription factors, RORγt and RORα, have recently been suggested as potential drugs for inhibiting Th17 development and treating CNS inflammatory diseases. However, inhibiting STAT3 pathways completely blocks Th17 development, as well as, prevents trafficking of inflammatory cells into CNS tissues, making STAT3 a more attractive therapeutic target. Thus, use of ORLL-NIH001 to target the STAT3 transcription factor, thereby antagonizing Th17 expansion and expression of proteins that mediate T cell chemotaxis, provides an attractive new therapeutic approach for treatment of posterior uveitis and other CNS autoimmune diseases mediated by Th17 cells.

## Introduction

T-helper cells are immune cells that mediate adaptive immunity in vertebrates and are comprised of 4 major subtypes, Th1, Th2, Th17 and Treg [1], [2], [3]. In comparison to other T-helper subsets, IL-17-producing T cells (Th17) are present in very low amounts in human blood but become highly elevated during chronic inflammation and are implicated in the pathology of several autoimmune diseases and chronic inflammatory disorders [4]. Th17 are therefore the subjects of intense research because they are potential drug targets for treating these disorders [5], [6], [7]. The differentiation of naïve CD4^+^ T cells towards the Th17 developmental pathway is promoted by IL-6 and TGF-β and mediated through activation of STAT3 pathways and Th17 lineage-specific transcription factors, RORα and RORγt [1], [3], [8]. Loss of STAT3 or RORγt expression abrogates Th17 differentiation and inhibits the production of cytokines secreted by Th17 cells [9]. In line with their role in Th17 differentiation, STAT3 and RORγt are attractive targets for treating autoimmune diseases such as uveitis, multiple sclerosis and inflammatory bowel disease.

Human uveitic diseases are estimated to be the cause of about 10% of severe visual loss in the United States and current understanding of the pathophysiology of uveitis derives largely from study of experimental autoimmune uveitis (EAU), a mouse model that shares essential features with human uveitis [10], [11]. Analysis of the recruitment of T cells from peripheral lymphoid tissues into the retina during EAU, revealed tremendous increase of Th17 cells in the blood, lymph nodes and retina of mice at onset and peak of the disease [4], [12]. However, their levels decline at late stages associated with recovery from acute uveitis [4], [12]. Treatment with anti-IL-17 antibodies ameliorated the disease, underscoring the involvement of Th17 cells in EAU pathology [4], [12]. Consistent with the role of Th17 in etiology of uveitis, mice with targeted deletion of STAT3 in the CD4^+^ T cell compartment (CD4-STAT3KO) are resistant to development of EAU [12]. CD4-STAT3KO mice are also resistant to experimental autoimmune encephalomyelitis (EAE), an animal model of human multiple sclerosis, further underscoring requirement of STAT3 pathway in CNS inflammatory diseases [13]. In EAU, significant numbers of the Th17 cells also express IFN-γ (Th17-DP) [4], [12]. These double expressors are absent in CD4-STAT3KO mice [12], [14], [15], indicating that they are also regulated by STAT3 and raising the intriguing possibility that uveitis maybe mediated not only by Th17 but also by Th17-DP cells. Requirement of STAT3 for generation of Th17 and Th17-DP cells also suggest that the STAT3 pathway is a potential therapeutic target that may be used to prevent or mitigate uveitis.

In this study, we induced EAU in B10.A mouse strain by immunization with interphotoreceptor-retinoid-binding protein (IRBP) [11]. We show here that a synthetic small molecule (ORLL-NIH001) that inhibits STAT3 reduced the severity of EAU by inhibiting Th17 expansion and inhibiting the expression of proteins that mediate the recruitment of inflammatory cells into the neuroretina. We further show that ORLL-NIH001 blocked the transcriptional activity of STAT3 and antagonized the expansion of human Th17 cells, providing suggestive evidence that it can be used to treat uveitis and other chronic inflammatory diseases caused by Th17 and Th17-DP cells.

## Materials and Methods

### Normal human subjects

Blood samples were obtained after IRB approval and consent from 10 normal human subjects that voluntarily donated blood to the National Institutes of Health (NIH) Blood Bank administered by the NIH Department of Transfusion Medicine. Each blood donor reads and signs consent form approved by the NIH ethics and intramural institute review boards. NIH Department of Transfusion Medicine adheres to the Declaration of Helsinki on research on human subjects. PBMCs were isolated from heparin treated whole blood by density gradient centrifugation and CD4^+^ T cells were isolated using the Rosette Sep kit (Stem Cell Tech, VA, Canada). PBMC or CD4^+^ cells were stimulated for 4 days with human anti-CD3 Abs (1 µg/ml), anti-CD28 Abs (3 µg/ml) in medium containing ORLL-NIH001 or vehicle. In some experiments exogenous IL-6 (10 ng/ml) (R&D Systems Minneapolis, MN) was added to medium.

### Mice

C57BL/6 and B10.A mice (6–8 weeks old) were from Jackson Laboratory (Bar Harbor, ME). Mice with conditional deletion of STAT3 in CD4 T cell compartment (CD4-STAT3KO) have been described [12]. Animal care and use was in compliance with Association for Assessment and Accreditation of Laboratory Animal Care, International (AAALAC) and the NIH Intramural Animal Care & Use Program guidelines. This study was approved by the National Eye Institute/NIH Animal Care and Use Committee under the Animal Study Proposal (ASP) # NEI-597 approved on October 25, 2010).

### Drug: ORLL-NIH001

ORLL-NIH001 is a synthetic small chemical compound developed by Orchid Research Laboratories Limited, Chennai, India. ORLL-NIH001 is a small molecule of 406-kDa with 98% purity as determined by HPLC and has been shown in various pre-clinical *in vitro* and *in vivo* models of oncology to be an inhibitor of pSTAT3. In pilot studies several doses (1 mg, 5 mg, 10 mg, 30 mg, 50 mg/kg/mouse) were administered intravenously to IRBP-immunized mice: 10 mg/kg/mouse was found to be most efficacious dose. Drug was solubilized in Vehicle [aqueous mixture of Hydroxyl propyl β-cylodextrin (0.2 g/ml), succinic acid (0.1 g/ml)].

### Induction of EAU and Histology

EAU was induced by active immunization with bovine interphotoreceptor retinoid-binding protein (IRBP), using 150 µg for C57BL/6 mice and 50 µg for B10.A mice and human IRBP peptide, amino acid residues 1–20, (150 µg for C57BL/6 mice), in a 0.2 ml emulsion (1∶1 v/v with complete Freund's adjuvant (CFA) containing mycobacterium tuberculosis strain H37RA (2.5 mg/ml). Mice also received Bordetella pertussis toxin (0.2 µg/mouse) concurrent with immunization. Mice were divided into 2 treatment groups. Mice in Protocol 1, received the drug starting 1 day prior to immunization with IRBP/CFA (day −1) and every other day thereafter until day 17 after immunization. Mice in Protocol 2 began receiving the drug 5 days post-immunization and every other day thereafter until day 17 post-immunization. Mice were sacrificed on day 18 post immunization, a time point that coincides with peak EAU in mice [12]. Eyes for histological EAU evaluation were harvested 18 days post-immunization, fixed in 10% buffered formalin and serially sectioned in the vertical pupillary-optic nerve plane. Sections were stained with hematoxylin and eosin (H&E) and EAU was scored in a masked fashion from 0 to 4 according to the histopathologic grading method developed previously for murine EAU by Chan et al [16]. Severity of EAU was scored according to the following criteria: 0, no sign of infiltrate; 0.5, focal non-granulomatous, monocytic infiltration in the choroid, ciliary body and retina; 1, retinal perivascular infiltration and monocytic infiltration in the vitreous; 2, granuloma in uvea, presence of retinal vasculitis, photoreceptor folds, serous detachment and loss of photoreceptor; 3 or 4, presence of Dalen-Fuchs nodules (granuloma at the level of the retinal pigmented epithelium) and subretinal neovascularization and depending on the number and size of the lesions. For analysis of T cells that infiltrated the retina during EAU, eyes were dissected and retinas were minced and incubated shaking at 37°C for 60 min in RPMI-1640 media containing 0.5 mg/ml of collagenase type D (Worthington, New Jersey). Released cells were subjected to flow cytometric analysis using appropriately labeled mAbs specific to relevant mouse T cell surface marker.

### Analysis of CD4^+^ T-helper cells

Freshly isolated T cells or IRBP-stimulated T cells from blood, spleen, lymph nodes (LN) or retina were analyzed for expression of inflammatory proteins by FACS or the intracellular cytokine expression assay as described [17]. In some experiments naïve T cells were activated in plate-bound anti-CD3 Abs (10 µg/ml) and soluble anti-CD28 Abs (3 µg/ml) without exogenous cytokines or Abs (Th0) or under Th17 [anti-CD3/CD28 Abs, IL-6 (10 ng/ml), TGF-β1 (2 ng/ml), anti-IFN-γ Abs (10 µg/ml), anti-IL-4 Abs (10 µg/ml)] polarization condition. For intracellular cytokine detection, cells were re-stimulated for 5 h with PMA (20 ng/ml)/ionomycin (1 µM). Golgi-stop was added in the last hour and intracellular cytokine staining was performed using BD Biosciences Cytofix/Cytoperm kit (BD Pharmingen, San Diego, CA). FACS analysis was performed on a Becton-Dickinson FACSCalibur using labeled monoclonal and corresponding isotype control Abs (PharMingen). Detection of intracellular pSTAT proteins was as described by [18] with fixation of the cells in 1.6% formaldehyde and permeabilized in 100% methanol. Fluorochrome-conjugated pSTAT1, −3, and −5 antibodies were purchased from BD-Bioscience.

### Lymphocyte proliferation assay

T cells were cultured for 4 d in quintuplet cultures containing anti-CD3/CD28 Abs and CFSE (carboxyfluorescein succinimidyl ester). CFSE dilution assay was performed using a commercially available CFSE Cell Proliferation Kit (Molecular Probes, Inc., Eugene, OR). After 36 h, some cultures were pulsed with ^3^H-thymidine (0.5 µCi/10 µl/well) for 12 additional hours and analyzed. The ^3^H-thymidine incorporation assay data are mean CPM ± S.E. of responses of five replicate cultures. CFSE dilution assay and ^3^H-Thymidine incorporation were performed on different samples.

### Western blotting analysis

Preparation of whole cell lysates and immunodetection were performed as described [19]. Samples (20 µg/lane) were fractionated on 4–20% gradient SDS-PAGE, and antibodies used were: pSTAT1, pSTAT3, pSTAT5 (Cell Signaling Technology) and β-Actin (Santa Cruz Biotechnology, Santa Cruz, CA). Preimmune serum was used in parallel as controls and signals were detected with HRP-conjugated secondary F(ab')_2_ Ab (Zymed Laboratories) using the ECL-PLUS system (Amersham, Arlington Heights, IL). Some blots were analyzed with the NIH Image Quant program. Each band was normalized to corresponding β-actin band and expressed in arbitrary relative protein expression units.

### Statistical analysis

Statistical analyses were performed by independent two-tailed Student's t test. Probability values of ≤0.05 were considered statistically significant. The data are presented as mean+SD.

## Results

### STAT3 signal is required for recruitment of T cells into retina and development of EAU

**Figure 1:**
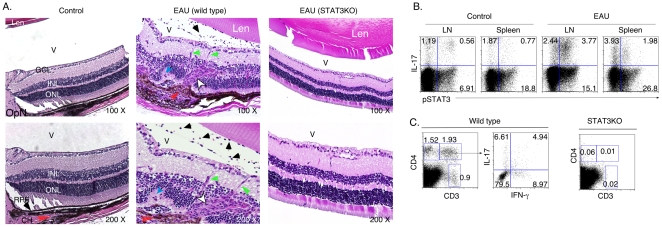
STAT3-deficient T cells could not traffic into the retina nor induce EAU. (A) WT mice or mice with targeted deletion of STAT3 in T cells (CD4-STAT3KO) were immunized with IRBP in CFA and their eyes were enucleated 21 days post-immunization and histological sections through the retina were stained with H&E. Black arrows indicate presence of inflammatory cells in the vitreous (V) and blue arrows depict pathologic foci characterized by the presence of retinal folds and hemorrhage. Red arrow, choroiditis; White arrow, granuloma; green arrow, retinal vasculitis; OpN, optic nerve; V, vitreous; GCL, ganglion cell layer; INL, inner nuclear layer; ONL, outer nuclear layer. (B) Freshly isolated cells from the draining lymph nodes or spleen of WT unimmunized WT control or EAU mice were analyzed by the intracellular cytokine assay. Plots were gated on CD4^+^ T cells and numbers in quadrants indicate percentage of CD4^+^ T cells expressing IL-17 and/or activated STAT3 (pSTAT3). (C) WT or CD4-STAT3KO mice were immunized with IRBP in CFA and T cells present in retina on day-21 post-immunization were detected and quantified by flow cytometry. Numbers in quadrants indicate percentage of CD3^+^ or CD4^+^ T cells or CD4^+^ T cells expressing IL-17 and/or IFN-γ. Data presented are representative of at least 3 independent experiments.

EAU was induced in WT or CD4-STAT3KO by immunization with IRBP in CFA, and consistent with previous studies, all WT mice developed severe EAU characterized by targeted destruction of photoreceptor cells [11] while none of the CD4-STAT3KO developed EAU (Fig. 1A). Intracellular cytokine staining analysis of the levels of STAT3 activation in the lymph nodes (LN) and spleen revealed substantial increase in pSTAT3 in mice with EAU compared to control mice (Fig. 1B). The positive correlation between high levels of pSTAT3 and increase of Th17 cells in the LN and spleen underscore the involvement of STAT3 signaling pathways for differentiation/expansion of Th17 cells that mediate EAU. We also observed substantial increase of CD4^+^ T cells in the retina of WT mice and the CD4^+^ T cell repertoire was characterized by the presence of Th17, Th1 and Th17-DP cells in the retina (Fig. 1C). In contrast, CD4^+^ T cells were barely detectable in the retina of IRBP-immunized CD4-STAT3KO mice. Taken together, these results suggest that STAT3 signaling is required for recruitment of T cells into retina and development of EAU and that drugs that block STAT3 signal transduction pathway may be useful in mitigating uveitis.

### A STAT3 inhibitor chemical (ORLL-NIH001) conferred protection from severe EAU

**Figure 2:**
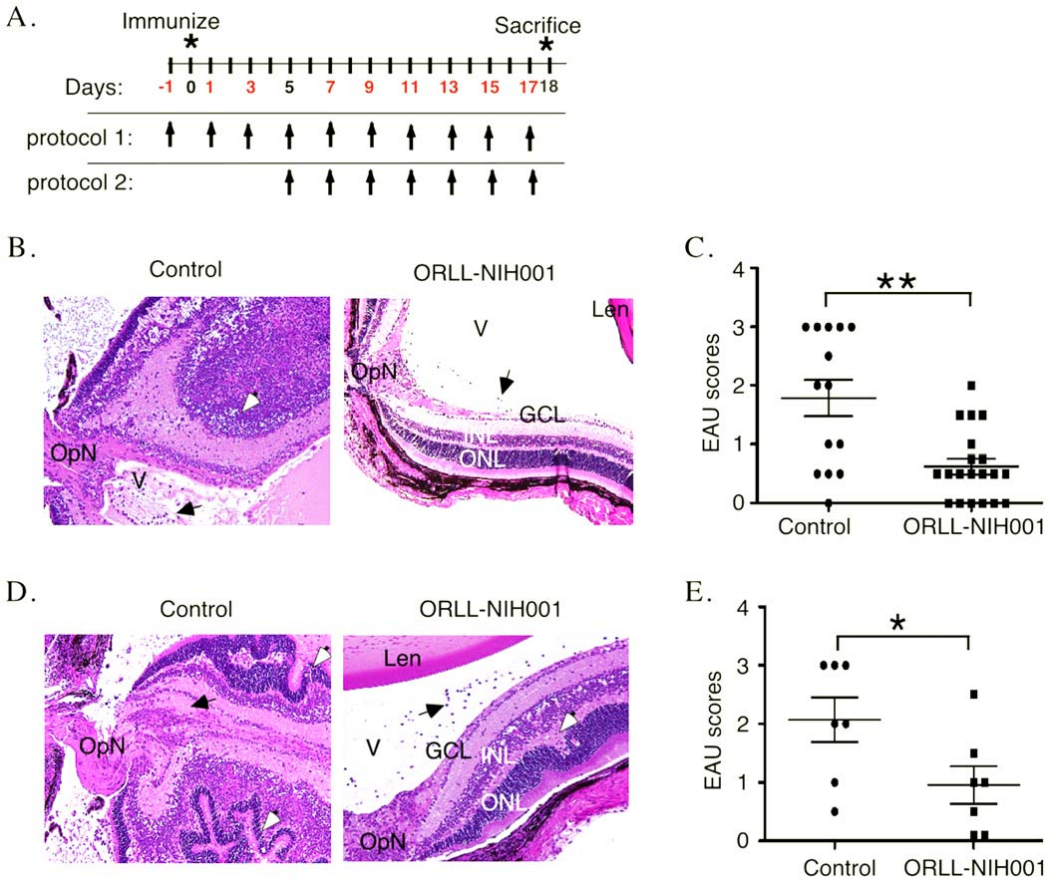
ORLL-NIH001 conferred protection from development of severe EAU. (A) Schematic depiction of immunization protocols used for EAU induction in B10.A mice. Mice treated under Protocol 1 received injections of ORLL-NIH001, starting 1 day before immunization (day −1) and on every other day thereafter until day-17 post-immunization. Protocol 2: mice began receiving the drug on day-5 post-immunization. Mouse eyes were harvested, fixed, embedded in paraffin and stained with H&E. (B, D) Histological analysis showing: inflammatory cells in the vitreous (black arrows), retinal folds (white arrows). OpN, optic nerve; V, vitreous; GCL, ganglion cell layer; INL, inner nuclear layer; ONL, outer nuclear layer. (C, E) EAU scores were calculated for vehicle-treated (control) or ORLL-NIH001-treated mice based on and histology. Similar results were obtained in mice treated under Protocol 1 (B, C) or Protocol 2 (D, E) and data presented are representative of at least 3 independent experiments.

The studies showing that mice with targeted deletion of STAT3 in T cells could not develop EAU [12] or EAE [12], [13] provided the requisite proof-of-principle experiments that spurred interest in the use of STAT3 antagonist as potential drugs for inhibiting Th17-mediated inflammatory diseases. In this study, we examined whether ORLL-NIH001, an inhibitor of STAT3, would be efficacious in mitigating uveitis. We induced EAU in B10.A mice by active immunization with IRBP and the mice were treated with varying doses of ORLL-NIH001, ranging from 1 mg/kg/mouse to 50 mg/kg/mouse per day. These pilot studies established that administration of 10 mg/kg/mouse by intravenous (i.v.) was most efficacious. The experimental design is depicted in Figure 2A. Aim of Protocol 1 study was to determine whether the drug could prevent development of EAU while Protocol 2 assessed whether the drug could reduce severity of ongoing disease. In Protocol 1, 36 mice (12 mice/experiment) received the drug at the times indicated (Fig. 2A), starting 1 day before immunization (day −1). In Protocol 2, each of 36 mice (12 mice/group) received the drug, starting on day 5 post-immunization and then every other day until day 17 post-immunization. For both protocols, age-match control mice were also immunized but they did not receive the drug. We show here that EAU was very severe in the B10.A mouse as indicated by the massive infiltration of inflammatory cells into the retina, vitreous and optic nerve (Fig. 2B; left panel). On the other hand, we observed dramatic reduction in the numbers of cells infiltrating the retina of mice treated with the drug (Fig. 2B; right panel) and the decrease in inflammation in drug-treated mice coincided with lower EAU scores (Fig. 2C). Similar to mice treated under Protocol 1, mice that began receiving the drug 5 days after T cell priming (Protocol 2) were also protected from developing severe EAU as indicated by reduction of inflammatory cells in their eyes (Fig. 2D; right panel) and significant reduction in their EAU scores (Fig. 2E). However, reduction of EAU severity was more dramatic in mice treated under Protocol 1. These results indicate that while the drug did not prevent the initiation of uveitis, it was effective at reducing the severity of ongoing disease.

### ORLL-NIH001 inhibited the expansion of uveitogenic Th17 cells

**Figure 3:**
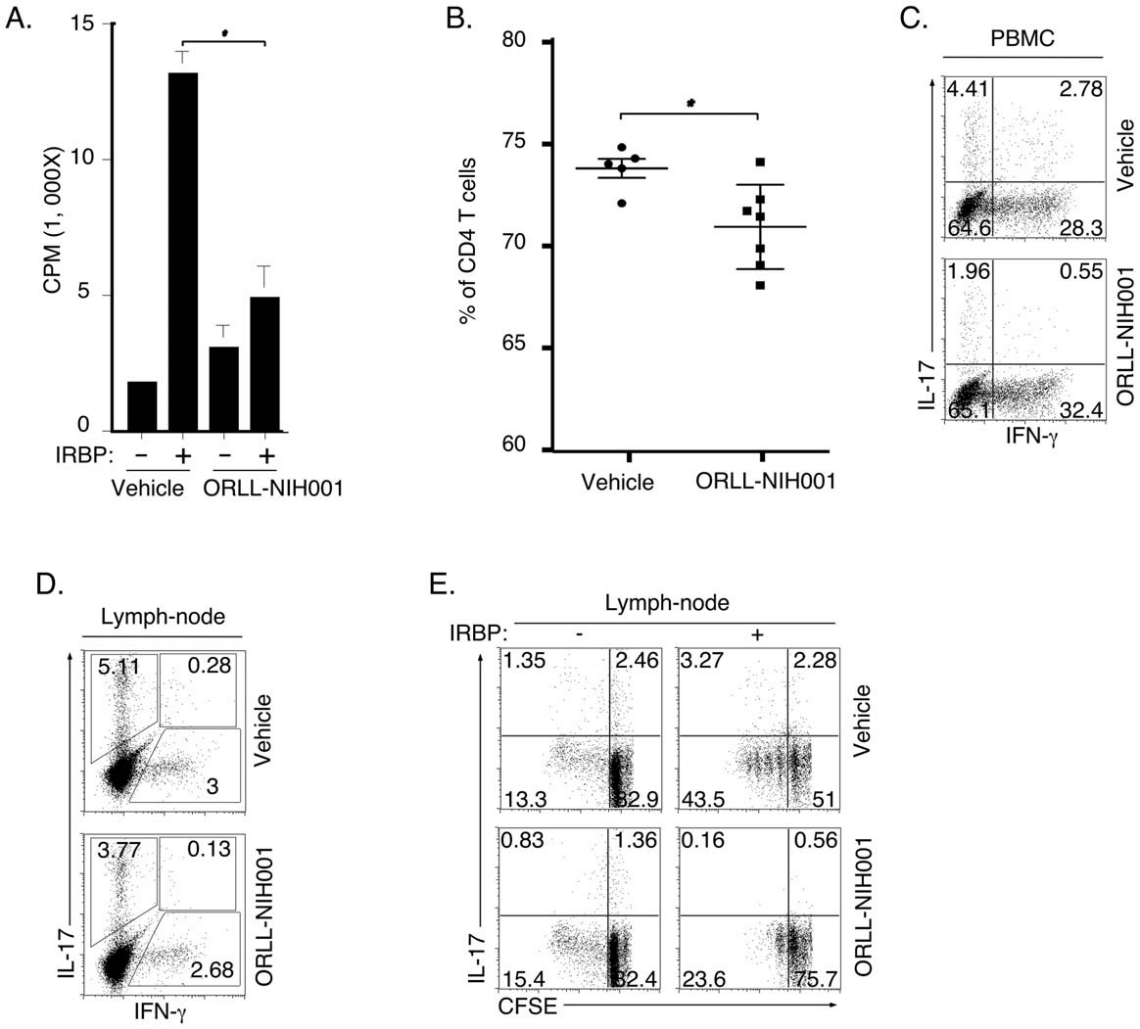
ORLL-NIH001 inhibited expansion of mouse uveitogenic Th17 cells. (A) Draining LN cells from vehicle-treated (control) or drug-treated mice (Protocol 1) were re-stimulated *in vitro* with IRBP for 3 days and effects of ORLL-NIH001 was assessed by Thymidine incorporation assay. Freshly isolated PBMC (B, C) was isolated from individual mice 4 days post-immunization. The levels of CD3^+^CD4^+^ T cells were quantified using FACS (B) and the number of cytokine-expressing T cells was assessed by intracellular cytokine assay (C). (D, E) Freshly isolated lymph node cells from vehicle or drug-treated mice were re-stimulated ex vivo with IRBP for 3 days and then analyzed by the intracellular cytokine assay. CFSE was added to some cultures (E). Plots were gated on CD3^+^ T cells and numbers in quadrants indicate percent of CD4^+^ T cells expressing IL-17 and/or IFN-γ. Data presented are representative of at least 3 independent experiments.

To characterize the mechanism by which ORLL-NIH001 inhibited EAU, we isolated draining LN cells from vehicle-treated (control) or drug-treated mice on day-18 post-immunization (Protocol 1) and re-stimulated the cells *in vitro* with IRBP for 4 days. Thymidine incorporation assay performed on the cells revealed that ORLL-NIH001 significantly inhibited proliferation of the IRBP-responsive CD4^+^ T cells; the proliferative capacity of drug-treated cells was decreased by more than three-folds compared to cells from mice treated with vehicle alone (Fig. 3A). To further examine effects of the drug before onset of disease, we isolated PBMC from individual mice 4 days post-immunization and quantified the relative levels of *in vivo* circulating CD4^+^ T cells in the vehicle- or drug-treated mice. FACS (Fluorescence-Activated Cell Sorting) analysis revealed significantly lower numbers of CD4^+^ T cells in their peripheral blood in the drug-treated mice even before the onset of EAU (Fig. 3B), suggesting that ORLL-NIH001 induced reduction in the numbers of activated IRBP-primed uveitogenic T cells that exit the LN and enter the blood. Analysis of the relative abundance of Th1 and Th17 cells among the peripheral blood CD4^+^ T cell population further revealed that the drug-treated mice contained substantially reduced levels of Th17 cells compared to control mice treated with vehicle alone (Fig. 3C). Interestingly, the inhibitory effects of ORLL-NIH001 were most dramatic on Th17-DP (compare 2.78% to 0.55%) (Fig.3C). On the other hand, the drug had marginal effects on the levels of Th1 cells in the PBMC (Fig. 3C) or draining LN (Fig. 3D), regardless of whether the mice were treated under Protocol 1 or 2. CFSE analysis of the IRBP-stimulated LN cells also revealed marked reduction in the level of resting or proliferating IL-17-expressing T cells in the drug treated mice, further underscoring effects of ORLL-NIH001 on Th17 cells (Fig. 3E).

### Inhibition of Th17 expansion by ORLL-NIH001 is dose-dependent

**Figure 4:**
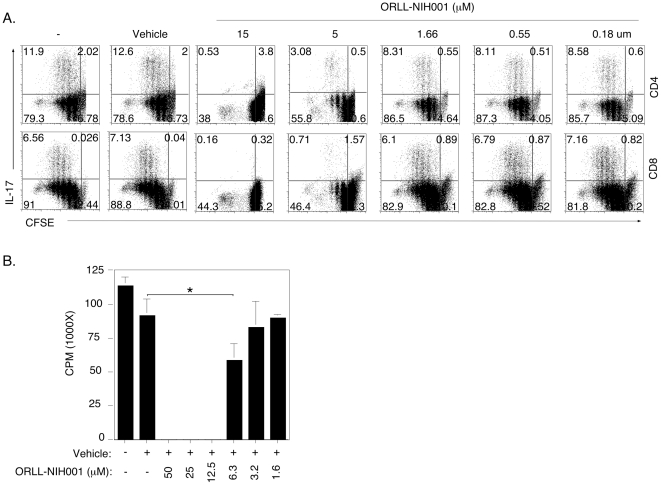
ORLL-NIH001 inhibits expansion of Th17 cells in a dose dependent manner. (A, B) Naïve CD4^+^ and CD8^+^ T cells were labeled with CFSE, stimulated for 4 days with anti-CD3/anti-CD28 under Th17 polarization condition in medium containing vehicle alone or different doses of ORLL-NIH001 (A). Numbers in the quadrants indicate percent of proliferating or non-proliferating CD4^+^ or CD8+ T cells expressing IL-17. (B) Proliferation of cells in cultures without CFSE was quantified by the Thymidine incorporation assay. Data presented are representative of at least 3 independent experiments.

To further characterize effects of ORLL-NIH001 on CD4^+^ and CD8^+^ T lymphocytes, we isolated naïve CD4^+^ or CD8^+^ T cells from B10A mice, labeled the cells with CFSE and then cultured them for 4 days under Th17 or Tc17 polarization condition in medium containing vehicle alone or different doses of ORLL-NIH001. We show here that the drug inhibited the proliferation of IL-17-expressing CD4^+^ and CD8^+^ T cells in a dose-dependent manner (Fig. 4A). The dose-dependent inhibitory effects of ORLL-NIH001 were further confirmed by the Thymidine incorporation assay (Fig. 4B).

### STAT3-inhibitors inhibit expression proteins that mediate lymphocyte trafficking into the retina

**Figure 5:**
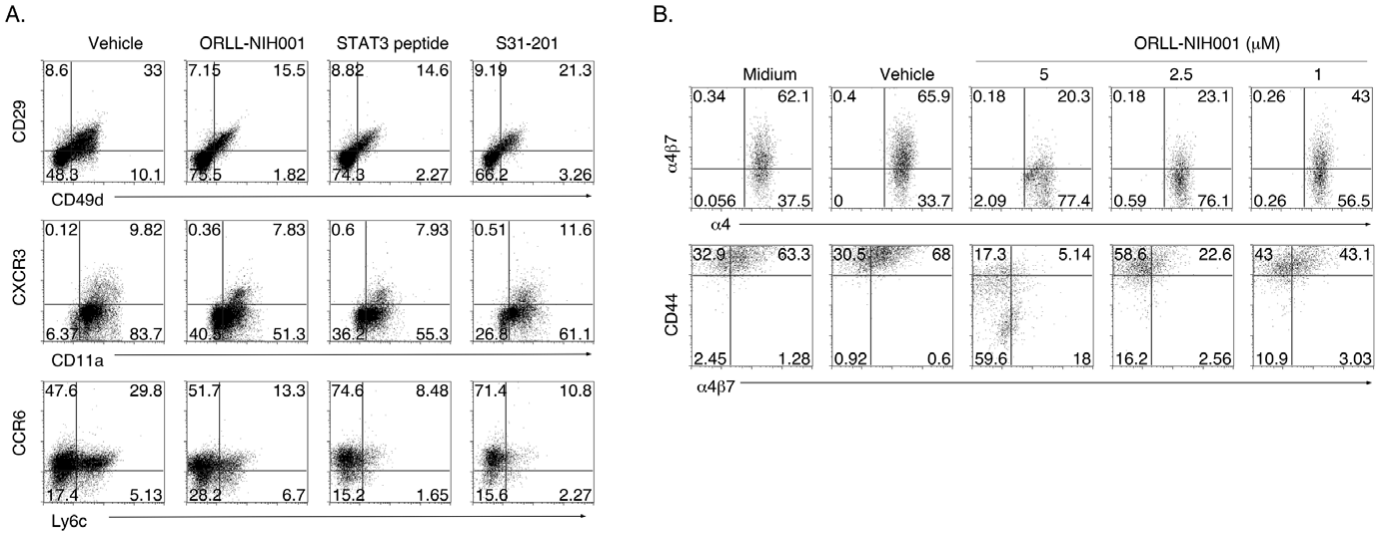
ORLL-NIH001 inhibited proteins that mediate lymphocyte trafficking into retina. (A, B) LN cells from mice with EAU were re-stimulated with IRBP in medium containing ORLL-NIH001 or commercially available STAT3 inhibitors. Expression or activation of integrins and chemokine receptors were analyzed by FACS. Plots were gated on CD4^+^ T cells and numbers in quadrants indicate percent of CD4^+^ T cells expressing CD11a, CD29, CD44, CD49d, CXCR3, CCR6, α4β7α4β1 or Ly6c. Data presented are representative of 3 independent experiments.

Integrins play important roles in the extravasation of lymphocytes into CNS tissues [20] and resistance of CD4-STAT3KO mice to EAU or EAE derives in part from defects in the expression of integrins that mediate trafficking of uveitogenic or encephalitogenic T-cells into the CNS during EAU or EAE [12]. We therefore examined whether reduction in inflammatory cells observed in the retina of mice treated with ORLL-NIH001 (Fig. 2), derived from the inhibition of integrins, adhesion molecules and/or chemokine receptors expression. Draining LN cells from mice with EAU were re-stimulated with IRBP and analyzed by FACS for the expression of molecules implicated in chemotaxis and homing of lymphocytes into CNS tissues. Compared to control mice, CD4^+^ T cells from drug-treated mice expressed much reduced levels of CD49d (integrin α4) and CD29 (integrin β1) and the activation VLA-4 (α4β1 was markedly inhibited by ORLL-NIH001 (Fig. 5A). IRBP-specific LN T cells from drug-treated mice also expressed much reduced levels of the Th1- and Th17-specific chemokine receptors, CXCR3 and CCR6 (Fig. 5A). These results were reproduced and validated by similar studies using two well-characterized and commercially available selective inhibitors of STAT3: (i) a cell-permeable phosphopeptide that inhibits STAT3 by binding to STAT3-SH2 domain (STAT3 peptide) (Calbiochem, La Jolla, CA); (ii) an amidosalicylic acid compound that selectively inhibits STAT3 activation and STAT3-dependent transcription (S31-201) (Calbiochem, La Jolla, CA). We further show that the expression of α4β7 integrin that promotes lymphocyte migration and CD44 that mediates leukocytes adhesion were also inhibited by ORLL-NIH001 in a dose dependent manner (Fig. 5B). These results suggest that ORLL-NIH001 mitigated EAU, in part, by inhibiting recruitment of uveitogenic T cells into the retina.

### ORLL-NIH001 inhibited the expansion of human Th17 cells

**Figure 6:**
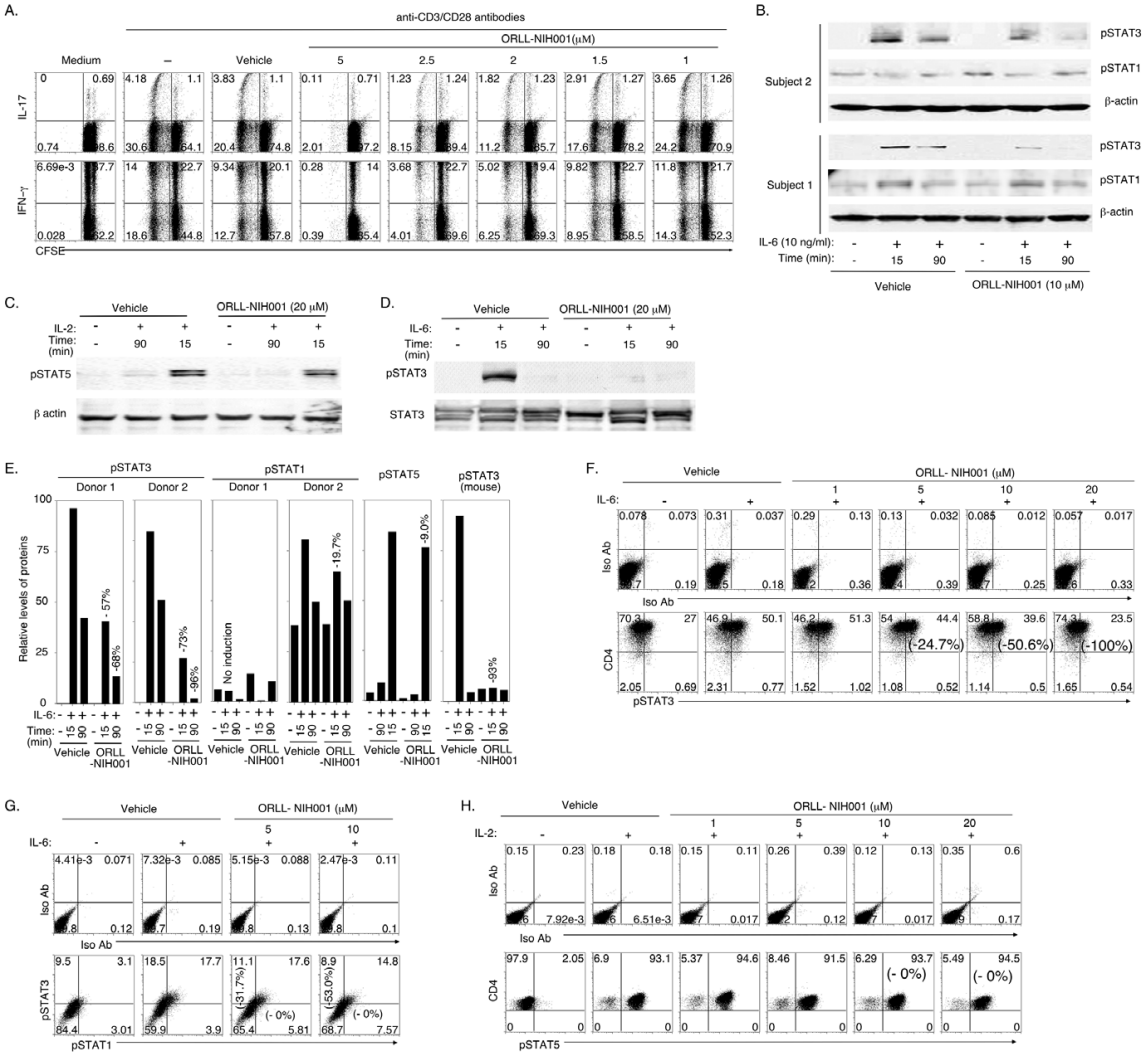
ORLL-NIH001 inhibited the expansion of human Th17 cells. (A) CD4^+^ T cells isolated from PBMC of healthy human subjects were labeled with CFSE and then stimulated with anti-CD3/CD28 for 4 days in medium containing vehicle alone or different doses of ORLL-NIH001. Numbers in the quadrants indicate percent of proliferating or non-proliferating T cells expressing IL-17 or IFN-γ. (B) Human PBMC were activated with anti-CD3/CD28 abs for 4 days, stimulated with IL-6 in serum-free medium containing vehicle or ORLL-NIH001. The cells were then analyzed for the expression of STAT3, pSTAT1 or β-actin by Western blotting. Activated human (C) or mouse (D–H) CD4 T cells were stimulated with IL-2 or IL-6 and expression of pSTAT1, pSTAT3 and pSTAT5 was detected by Western blotting (C, D, E) or flow cytometry (F, G, H). (E) Western blots (B, C, D) were analyzed using NIH Image Quant program and each band was normalized to corresponding β-actin band and expressed in arbitrary relative protein expression units. Results are representative of 3 independent experiments.

Our data showing that ORLL-NIH001 inhibited the recruitment of inflammatory cells into mouse retina and mitigated EAU raised the tantalizing question of whether it has similar inhibitory effects on human lymphocytes. To investigate whether ORLL-NIH001 can inhibit the expansion of human T cells we isolated T cells from peripheral blood of normal human volunteers, stimulated the cells with anti-CD3/anti-CD28 Abs for 4 days in medium containing the vehicle or varying doses of ORLL-NIH001. We show here that ORLL-NIH001 inhibited the proliferation of Th17 and Th1 cells (Fig. 6A). It is however of note that, the drug primarily targeted highly proliferating cells (Fig. 6A). Similar to the inhibitory effects of ORLL-NIH001 on mouse IRBP-specific T cells that mediated EAU, the suppression of human Th17 expansion by ORLL-NIH001 was dose-dependent (1 to 5 µM). To determine whether ORLL-NIH001 selectively targeted STAT3 pathway in human lymphocytes, CD4^+^ T cells from human PBMC were activated with anti-CD3/CD28 abs for 4 days, cultured in serum-free medium for 2 hours and then stimulated with IL-6 in medium containing vehicle alone or ORLL-NIH001 (10 µM). Consistent with its specificity for STAT3, ORLL-NIH001 inhibited STAT3 phosphorylation of human and mouse cells but had marginal effects on STAT1 or STAT5 activation (Fig. 6B–E). We further show by intracellular staining assay that the inhibitory effects of ORLL-NIH001 on STAT3 activation is dose-dependent (Fig. 6F, G). Similar to our Western blotting data, we did not observe substantial suppression of STAT1 or STAT5 activation (Fig. 6F–H).

## Discussion

Human uveitis is a diverse group of chronic intraocular inflammatory diseases that can lead to blindness and they include sympathetic ophthalmia, birdshot retinochoroidopathy, Behçet's disease, Vogt-Koyanagi-Harada syndrome, pars planitis, and ocular sarcoid [21], [22], [23]. Blinding uveitis is characterized by repeated cycles of remission and recurrent intraocular inflammation and although steroids and other anti-inflammatory drugs are effective therapy, renal toxicity or other adverse effects preclude prolonged usage [22], [24]. Involvement of Th17 and Th17-DP cells in mouse [12], [25] and human uveitis [4] suggest that they may be potential targets for treating uveitis. Given the essential role of STAT3 in Th17 cell differentiation and effector functions, proof-of-principle experiments showing that genetic ablation of STAT3 in T cells prevented development of EAU and EAE in mice [12], [13], provided impetus to develop drugs that target STAT3 pathways for treating uveitis and other autoimmune diseases mediated by Th17 cells.

In this study, we have shown that ORLL-NIH001, a small molecule that targets STAT3 pathways in T cells was effective in mitigating uveitis in mice. The drug-treated mice contained substantially reduced levels of Th17 cells compared to control mice treated with vehicle alone (Fig. 2). Interestingly, the inhibitory effects of ORLL-NIH001 on T cell expansion were most dramatic onTh17-DP and it is of note that a strong correlation has previously been noted between the expansion of the Th17-DP subtype in the blood, LN and retina and the development of uveitis in the mouse [4], [12]. These results are consistent with previous report showing that the resistance of CD4-STATKO mice to EAU or EAE development derived from failure to generate Th17 cells, marked reduction in the expression of activated α4/β1 integrins and inability of pathogenic Th17/Th1 cells to traffic into the retina or brain [12]. α4β1 and α4β7 integrins have been implicated in chronic demyelinating diseases [26], [27], [28], [29]. Recent studies have shown that α4β1 mediates the recruitment of immature dendritic cells across the blood-brain barrier [30] and is required for entry of encephalitogenic T cells into the CNS during EAE [31], [32]. On the other hand, how α4β7 contributes to EAE pathogenesis is not well understood, as ectopic expression of MAdCAM-1 at the blood-brain barrier failed to increase α4β7-mediated immune cell trafficking into the CNS during EAE [33]. Nonetheless, inhibiting the α4 subunit of the integrin heterodimers, α4β1 and α4β7, with the mab natalizumab has been found to be effective in the treatment of multiple sclerosis (MS) [34]. Although the involvement of α4β1 and α4β7 has not been established in EAU, the extensive distribution of adhesion molecules on the endothelium of the vessels in the retina and iris in patients with uveitis suggest their potential role in the development of the disease [35].

In this study, we observed that decrease in inflammatory cells in the eyes of mice treated with ORLL-NIH001 correlated with the down-regulation of activated α4β1 α4β7 CCR6 and CXCR3 and the inhibitory effects of ORLL-NIH001 derived in part from inhibiting the tyrosine phosphorylation of STAT3 in response to pro-inflammatory proteins. We have also shown that a cell-permeable phosphopeptide molecule, as well as, an amidosalicylic acid compound that bind to STAT3-SH2 domain inhibited STAT3 activation and the expression of CXCR3 and CCR6, suggesting that ORLL-NIH001 may in part ameliorate EAU by inhibiting STAT3-dependent transcription. Together, these results indicate that ORLL-NIH001 mitigates uveitis by 2 distinct mechanisms: It inhibits Th17 differentiation/expansion and prevents entry of inflammatory cells into the neuroretina. It is notable that EAU was markedly inhibited in mice that were treated with ORLL-NIH001 1 day before disease induction, as well as, mice that received the drug after establishment of the disease process, suggesting potential value of ORLL-NIH001 in treating pre-existing uveitis. Although delivery of ORLL-NIH001 by intravenous injection is effective for treating EAU, we are currently exploring alternative routes for delivery of the drug, such as subcutaneous injection, oral administration or direct injection of ORLL-NIH001 into the eye.

The pathway towards Th17 differentiation in mouse and humans are also regulated by two retinoic acid receptor-related orphan nuclear receptors, RORγt and RORα [36], [37], [38]. Loss of expression of these Th17 lineage transcription factors prevents differentiation of Th17 cells and development of Th17-mediated autoimmune diseases in mice [5]. In a recent report digoxin and its derivatives were found to inhibit mouse Th17 differentiation and ameliorate EAE [7]. In another report a synthetic ligand, SR1001, specific to RORγt and RORα exhibited similar inhibitory effects on Th17 development and EAE [6]. These reports, together with results presented in this study usher a new therapeutic approach for treatment of autoimmune diseases and the challenge is to develop non-toxic small molecule compounds that can effectively target Th17 transcription factors. Although all three molecules, ORLL-NIH001, SR1001 and the non-toxic digoxin derivates, ameliorate EAU or EAE by inhibiting Th17 differentiation and antagonizing IL-17 expression/maintenance of Th17 phenotype, ORLL-NIH001 has the additional benefit of also inhibiting the trafficking of inflammatory cells into the CNS tissues. ORLL-NIH001 was not cytotoxic to mouse or human cells at concentrations that inhibited EAU, making it suitable candidate drug for treatment of human autoinflammatory disease. However, as is the case with RORγt and RORα, STAT3 is expressed in other cell types and long-term treatment with any of these inhibitors of Th17 development may have unwanted side effects. Nonetheless, it is important to note that treatment for patients with relapsed or refractory posterior uveitis or panuveitis remains challenging and prolonged use standard anti-uveitic therapy is associated with significant risk of developing cataract and glaucoma [22], [24]. Thus, a small molecule such as ORLL-NIH001 that selectively target a critical transcription factor that plays critical roles in recruitment of inflammatory cells into the immune-privileged neuroretina offer an attractive alternative for treating these potentially blinding diseases.
